# Structure of a Stapled Peptide Antagonist Bound to Nutlin-Resistant Mdm2

**DOI:** 10.1371/journal.pone.0104914

**Published:** 2014-08-12

**Authors:** Sharon Min Qi Chee, Jantana Wongsantichon, Quah Soo Tng, Robert Robinson, Thomas L. Joseph, Chandra Verma, David P. Lane, Christopher J. Brown, Farid J. Ghadessy

**Affiliations:** 1 p53Lab, Agency for Science Technology and Research (A*STAR), Singapore, Singapore; 2 Institute of Molecular and Cellular Biology, A*STAR, Singapore, Singapore; 3 Bioinformatics Institute, A*STAR, Singapore, Singapore; 4 Department of Biological Sciences, National University of Singapore, Singapore, Singapore; 5 School of Biological Sciences, Nanyang Technological University, Singapore, Singapore; Rush University Medical Center, United States of America

## Abstract

As key negative regulator of the p53 tumour suppressor, Mdm2 is an attractive therapeutic target. Small molecules such as Nutlin have been developed to antagonise Mdm2, resulting in p53-dependent death of tumour cells. We have recently described a mutation in Mdm2 (M62A), which precludes binding of Nutlin, but not p53. This Nutlin-resistant variant is not, however, refractory to binding and inhibition by stapled peptide antagonists targeting the same region of Mdm2. A detailed understanding of how stapled peptides are recalcitrant to Mdm2 mutations conferring Nutlin-resistance will aid in the further development of potent Mdm2 antagonists. Here, we report the 2.00 Å crystal structure of a stapled peptide antagonist bound to Nutlin resistant Mdm2. The stapled peptide relies on an extended network of interactions along the hydrophobic binding cleft of Mdm2 for high affinity binding. Additionally, as seen in other stapled peptide structures, the hydrocarbon staple itself contributes to binding through favourable interactions with Mdm2. The structure highlights the intrinsic plasticity present in both Mdm2 and the hydrocarbon staple moiety, and can be used to guide future iterations of both small molecules and stapled peptides for improved antagonists of Mdm2.

## Introduction

Cell fate is primarily governed by the p53 tumour suppressor [1], [2]. Triggered by stresses such as DNA damage and hypoxia, p53 elicits numerous cellular outcomes including cell cycle arrest and cell death. Loss of p53 function, typically arising through point mutations is seen in 50% of all cancers [3], [4]. In malignancies with wild type p53 status, the activity of p53 is commonly attenuated through overexpression of Mdm2, a key negative regulator [5], [6]. Mdm2 both inhibits the transactivation function of p53 and selectively ubiquitinates p53, targeting it for proteosomal degradation [7]–[10]. Re-instatement of p53 effector functions has been demonstrated by inhibition of Mdm2 with both small molecule and peptide antagonists [11]–[14]. Nutlin-3a (hereafter termed Nutlin) is the proto-typical small molecule Mdm2 antagonist [15]. It competes with p53 for binding to an extended hydrophobic cleft in the N-terminal domain of Mdm2. Binding is achieved by recapitulating interactions of three key p53 amino acid side chains (F19, W23, L26), with discrete pockets lining the hydrophobic cleft. We have previously described the mutations M62A and Q24R in the N-terminal domain of Mdm2 that impart Nutlin-resistance by selectively reducing affinity for Nutlin but not p53 [16]. In the case of the M62A mutation, substitution of the methionine removes a key packing interface required by Nutlin, significantly impairing binding. Whilst M62 also contributes to the binding of p53 peptide (residues 15 to 29 of p53, Figure 1), loss of this residue is mitigated by an extended network of Van der Waals contacts distributed along the Mdm2 binding cleft [17].

**Figure 1:**
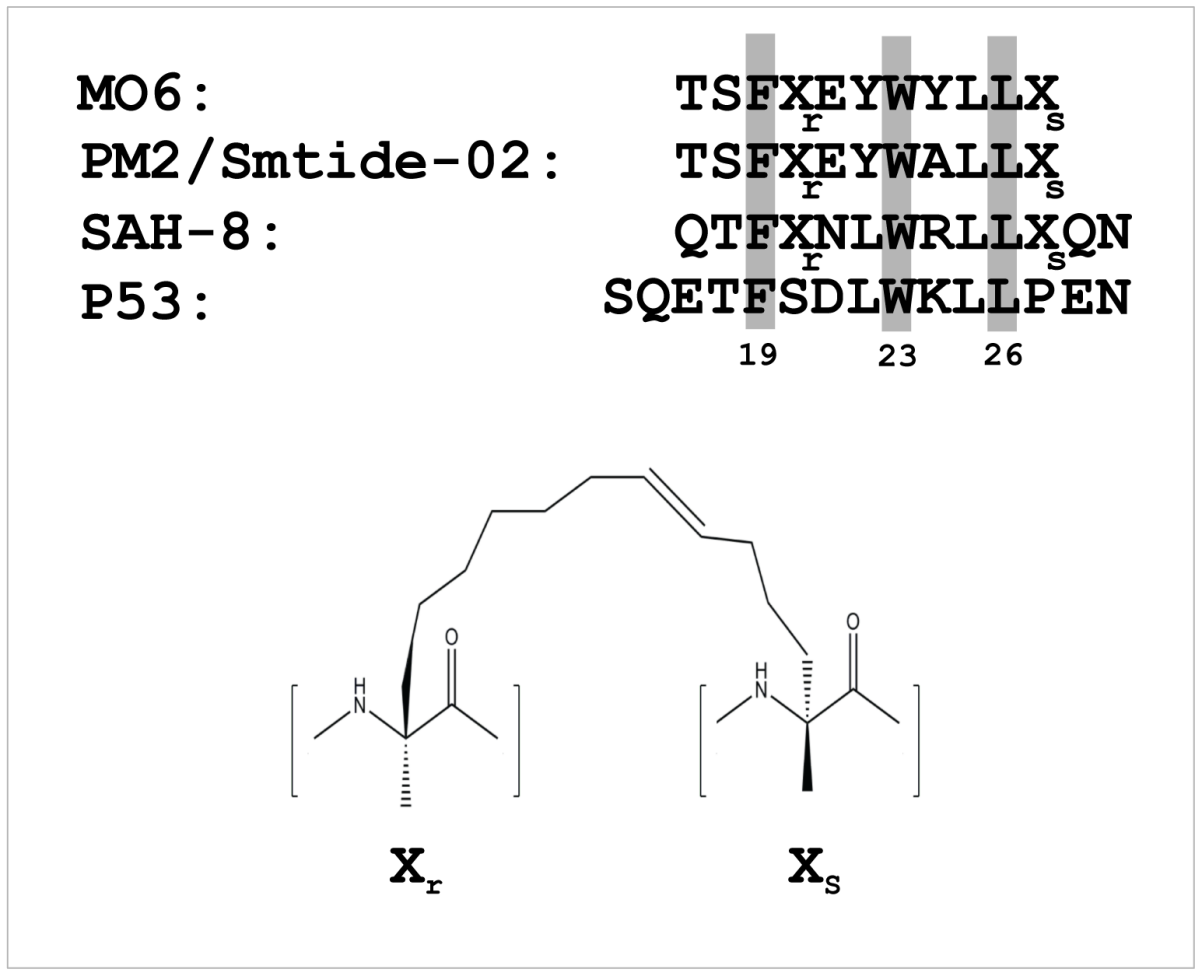
Sequence alignment of peptide ligands targeting the Mdm2 N-terminal domain. The critical interacting residues (F19, W23 and L26) in the p53 wild-type peptide and conserved in the indicated stapled peptides are shaded. The staple tethering site is denoted by ‘X’ and chemical structure of the staple moiety (adapted from [18]) is shown below.

We recently demonstrated that stapled peptide analogues of Nutlin targeting Mdm2 are able to bind and inhibit both wild type and the M62A/Q24R resistant variants in biophysical and cell-based assays [18], [19]. Stapled peptides comprise a covalent linkage bridging adjacent turns of an alpha helical peptide (the “staple”) [20]. By pre-stabilising favourably interacting conformer(s), the staple increases affinity by reducing the entropic penalty of binding. Furthermore, it imparts significant proteolytic stability, increased half-life *in vivo*, and in certain instances allows adjunct-free cellular uptake [21]–[23]. These ideal drug-like properties have seen stapled peptides entering clinical trials.

Here, we present the three dimensional crystal structure of a stapled peptide antagonist bound to Nutlin-resistant mutant Mdm2. The structure gives an insight into the remarkable plasticity of Mdm2 that facilitates binding of small molecule and peptide antagonists.

## Results and Discussion

### Characterisation of M06 stapled peptide

The Mdm2-binding stapled peptide used in this study, M06/sMTide-06, is a variant of the previously described PM2/sMTide-02 stapled peptide shown to bind mutant Mdm2 with high affinity [18]. The alanine residue at position 8 in PM2 is mutated to tyrosine in M06, with all other structural characteristics remaining identical (Figure 1). MO6 bound both wild type and Mdm2-M62A (residues 6–125) with similar affinities as determined by fluorescence polarisation (apparent K_d_s of 107.49±47.9 nM and 63.13±17.81 nM, respectively) (Figure S1 in File S1). M06 also displayed similar activity to PM2 in a T22 cell-based reporter assay measuring p53 activation (Figure S2 in File S1).

### Structure determination of M06 bound to Mdm2-M62A

**Figure 2:**
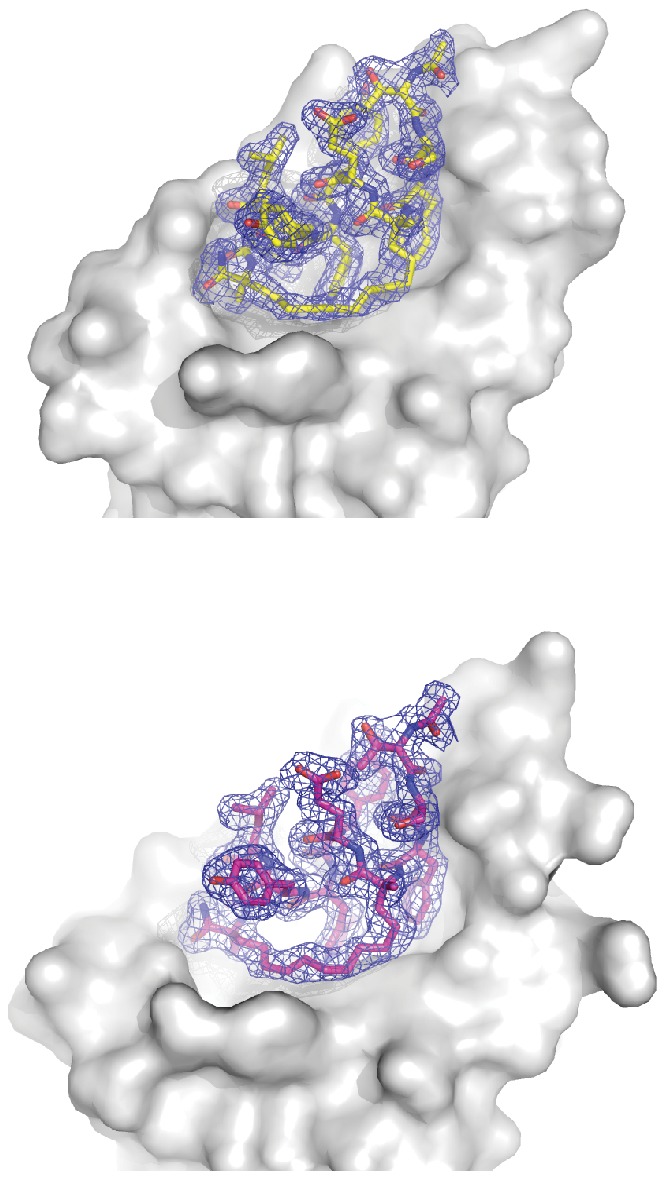
Crystallographic Unique Complexes of the M06 stapled peptide bound to the N-terminal p53 binding domain of Mdm2-M62A (6–125). The 2Fo-Fc electron density map, which has been contoured at 1.2 σ, clearly demonstrates the presence of the whole peptide bound to Mdm2.

**Figure 3:**
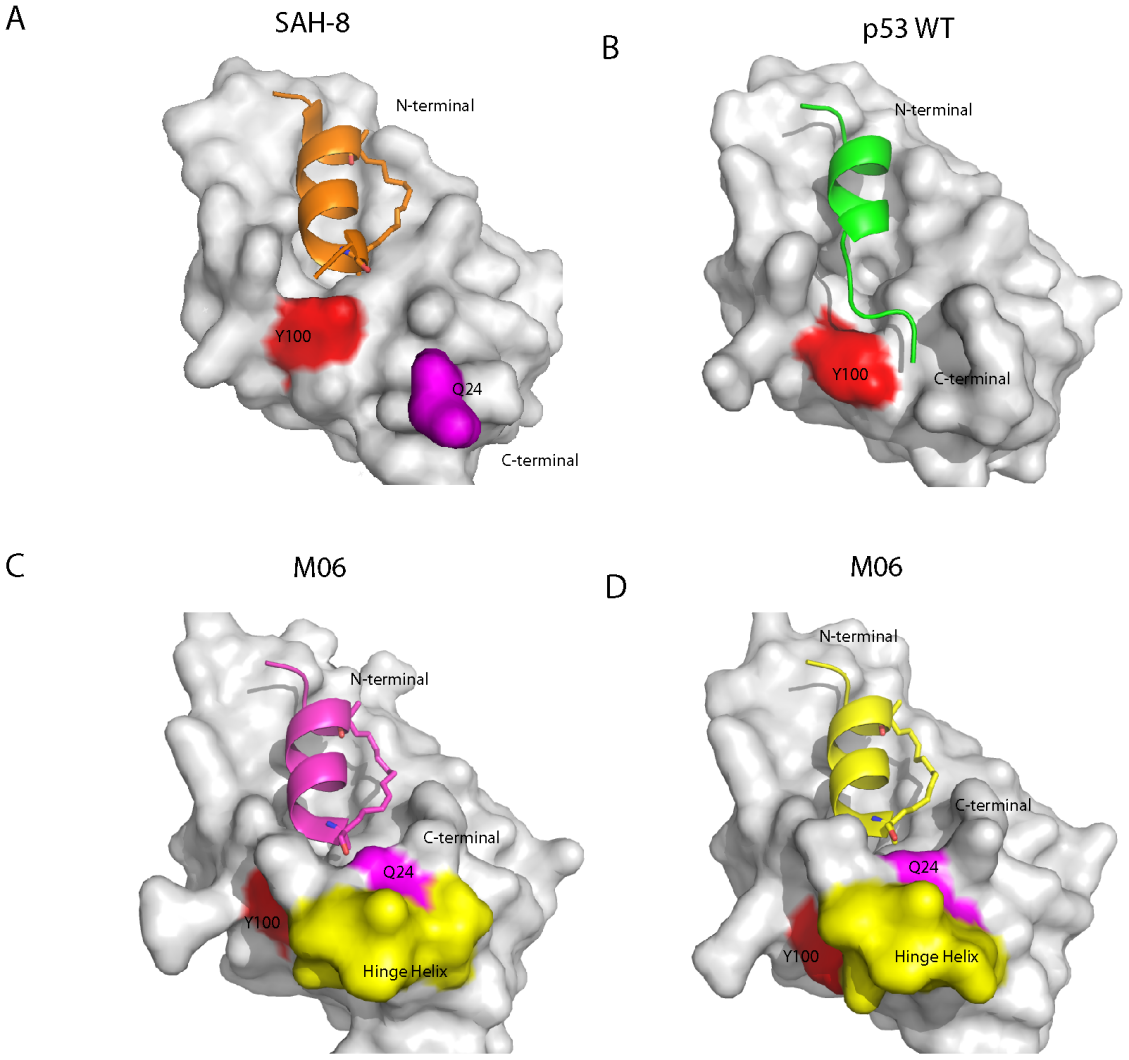
Mdm2-M62A (6–125) shows a high degree of conformational plasticity in the α2’ and ‘hinge’ helices capping the p53-binding pocket. A,B The binding pockets that form when Mdm2 interacts with SAH-8 (A) and p53 WT peptide (B) are much more open when compared to the binding pockets formed in the two complexes of Mdm2:M06 found in the asymmetric unit. The key residue that is involved in forming the bottom of the binding pocket in the Mdm2 complexes with SAH-8 (A) and p53 WT (B) is Y100 (shown in red) on the α2’ helix, where it either closes the binding site by orientating itself inward or out towards the solvent, respectively. C, D In contrast, the binding pockets formed in the two crystallographically unique M06 complexes are capped by the ‘hinge’ helix of the lid (residues 20–24, highlighted in yellow). The helix is stabilized in this conformation by a hydrogen bond between Q24 (magenta) and the amide cap at the C-terminal of M06.

The structure of M06 in complex with Mdm2-M62A was elucidated using X-ray crystallography, revealing 2 complexes in the asymmetric unit of the crystal (Table 1). The p53 peptide binding groove in both complexes was occupied by a single molecule of M06, where electron density in the 2Fo-Fc could be observed for the entirety of both molecules (Figure 2). M06 binds into a compact and deep groove on the Mdm2 surface. It also positions the conserved p53-derived Mdm2 interaction motif (F19, W23, L26) into an identical orientation compared to the crystal structures elucidated for Mdm2 in complex with linear peptides either derived from the p53 WT sequence (PDB: 1YCR) [17] or the PMI variant selected for high affinity (PDB: 3EQS) [24]. Several key differences can however be observed when the Mdm2-M62A:M06 structure is overlaid with these structures. The hydrocarbon staple in M06 induces greater helical character in the C-terminus of the peptide in comparison to PMI, where the C-terminal helical turns are more extended in character, whilst in the case of WT p53 the helicity of the peptide is broken at the proline position and the rest of the peptide forms an extended strand type structure (Figure 3). Additionally, in the Mdm2-M62A:M06 crystal structure the peptide binding groove is capped by the helical ‘hinge’ region of the lid (residues 20–24), where the C-terminus of the stapled peptide packs (Figure 3C, D). This helical region of Mdm2 interacts with the C-terminal amide group of M06 by forming hydrogen bonds via Q24. The capping of the Mdm2 peptide binding groove by the ‘hinge’ helix is not observed for the p53 WT peptide as it has a longer C-terminus than M06, which sterically excludes the possibility of this occurring (Figure 3B). In the case of the PMI:Mdm2 structure, the helical ‘hinge’ region is absent in the crystallization construct used. Interestingly, the greater helicity observed in the C-terminal helical turn of the stapled peptide results in significant alteration of the C-alpha position of L26. The L26 side chain packs into the peptide groove in an orientation that is very different from its conformations in the p53 WT and PMI unstapled peptides.

**Table 1:** Crystallographic data collection and refinement statistics.

Unit cell dimensions (Å)	a = 39.089 b = 65.677 c = 105.689, α = β = γ = 90°
Resolution (Å)	30.00 −1.97 (2.02–1.97)
Space group	P22_1_2_1_
Temp (K)	100
Redundancy	6.4 (5.8)
Collected Reflections	123113
Unique reflections	19576
R sym (%)	0.052 (0.340)
I/sigma	34.74 (4.87)
R factor (%)	20.05
R free (%)	23.68
RMS bonds (Å)	0.0049
RMS angle (°)	1.260
% Completeness	99.3 (95.3)
Wilson B-Factor (Å^2^)	29.8
Average B Value (Å^2^)	
Chain A	34.946
Chain B	35.750
Chain C (peptide)	27.106
Chain D (peptide)	28.446
Water	37.263
Number of Solvent Molecules	85
Ramachandran Data	
Favoured Region (%)	94.6
Additionally Allowed Region (%)	5.4
Generously Allowed Region (%)	0
Disallowed Region (%)	0

Parameters for highest resolution bin given in parenthesis.

**Figure 4:**
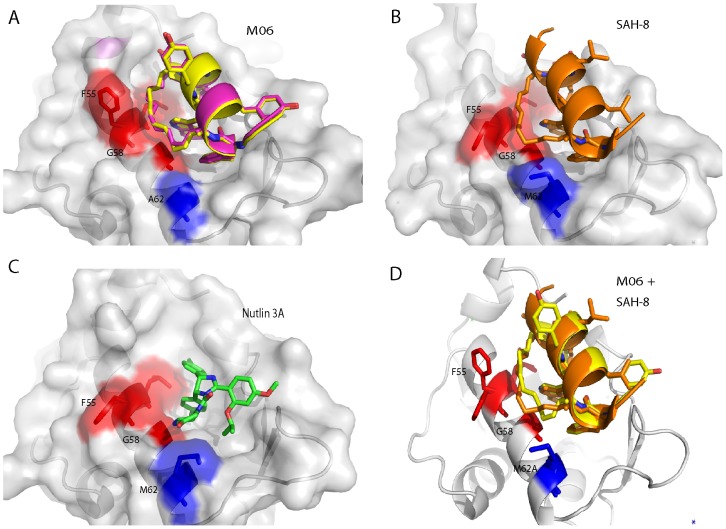
Comparison of Mdm2-M62A:M06 and Mdm2:SAH-8 structures. A, The staple in both Mdm2:M06 complexes, found in the asymmetric unit, packs against residues L54, F55 and more closely with G58 (shown in red) in the absence of M62. B, The conformation of the staple in SAH-8 differs subtly from that seen in the M06 complexes, with the staple in SAH-8 deformed by the interaction with M62 (shown in blue). C, The piperazinone ring of Nutlin forms critical contacts with M62, which are lost in the Nutlin resistant Mdm2. D, Overlay of the Mdm2-M62A:M06 complex with the Mdm2:SAH-8 complex, highlighting the conformational differences between the respective hydrocarbon staples in the presence and absence of M62. M06 is highlighted in yellow and SAH-8 in orange. The conformation of the F55 side-chain also changes, but this is primarily due to crystal packing effects.

The M06 structure was further compared to the crystal structures of a stapled p53 peptide (SAH-8) bound to Mdm2 (PDB 3V3B) [25] and to Nutlin bound to Mdm2 (PDB 4HGZ) [26] (Figure 4). The M06 stapled peptide forms interactions very similar to those made by the SAH-8 peptide, including the reorientation of the L26 side chain that appears to be associated with increased helicity. Apart from attenuating the binding of Nutlin, the M62A mutation also causes a significant change in the conformation of the aliphatic staple. In the SAH-8 structure the hydrocarbon chain packs predominantly against L54, F55, G58 and M62 (Figure 4B). The absence of the methionine side chain in Mdm2-M62A causes the conformation of the staple to change as it packs more closely against G58 (Figure 4A). The plasticity of the stapled peptide enables it to respond to the M62A mutation by making compensatory contacts. In stark contrast, the mutation results in the loss of several hydrophobic contacts between the piperazinone ring of Nutlin and the methionine (Figure 4C, Figure S3 in File S1). Nutlin is a much more rigid and smaller molecule, and cannot compensate for the loss of these contacts.

**Figure 5:**
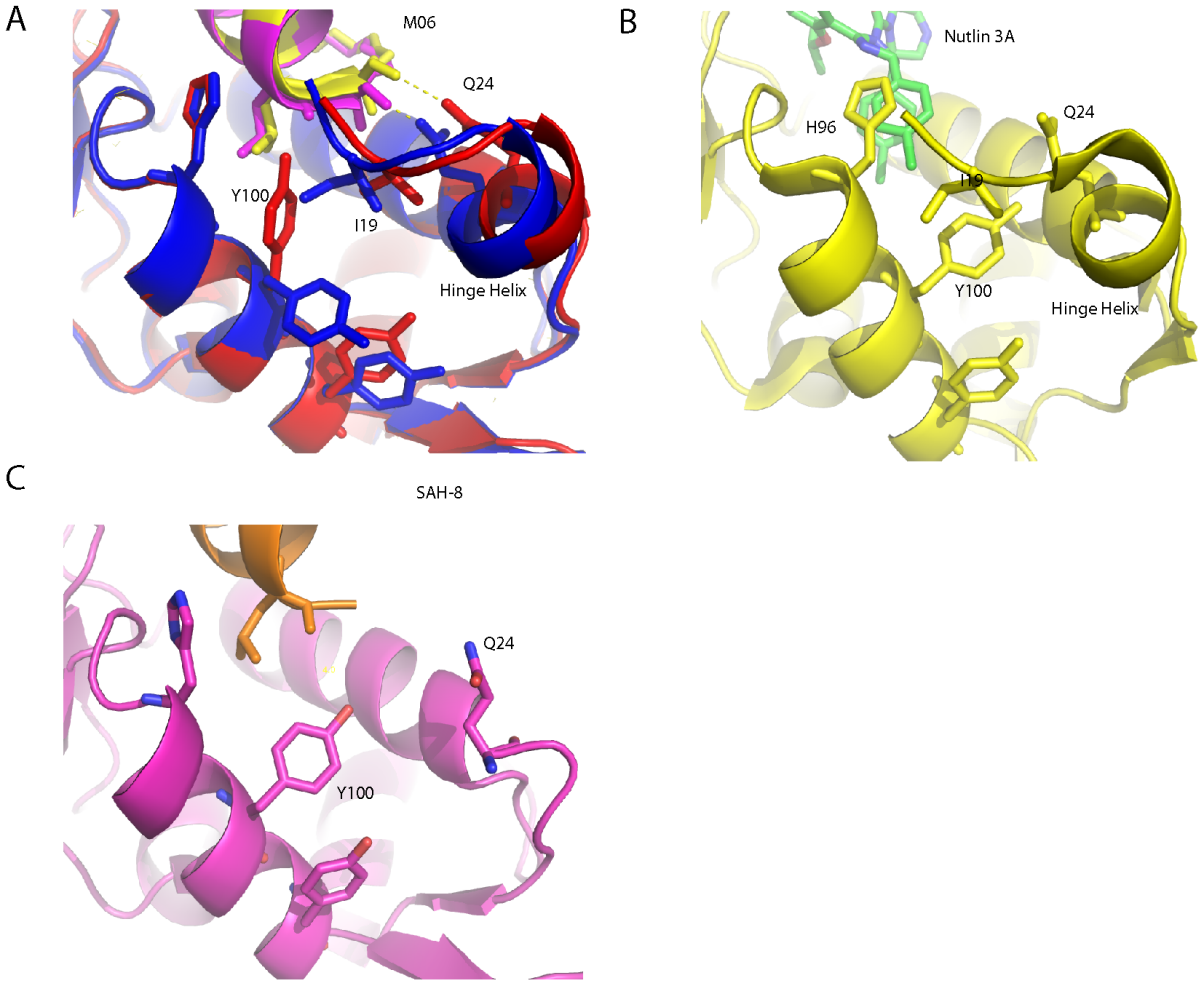
Differential orientation of Y100 in the Mdm2-M62A:M06 structure. A, Overlay of the two Mdm2:M06 complexes from the asymmetric unit of the crystal structure showing the packing interactions at the bottom of the binding pocket. In both complexes the ‘hinge’ helix caps the bottom of the pocket and is stabilized via a hydrogen bond between Q24 and M06. However, in the M06 complex highlighted in blue, the ’hinge’ helix swings further into the binding pocket and results in I19 preventing Y100 from orientating itself in towards the binding pocket. In contrast, the degree to which the helix moves into the pocket is less in the other M06 complex (shown in red). This results in Y100 forming a hydrogen bond to L26 and I19 packing alongside it to form the bottom of the binding pocket. B, In the Mdm2:Nutlin complex the ‘hinge’ helix caps the bottom of the binding pocket as seen in A. However, the Y100 adopts an intermediate position between the two orientations observed in the M06:Mdm2 complexes, with the I19 packing on top of it. In addition, H96 interacts with the chloro-benzyl moiety of Nutlin, causing the helix to swing into the peptide binding groove, inducing the pocket to narrow. C, Capping of the Mdm2 binding pocket by the ‘hinge’ helix does not occur for the SAH-8 complex due to steric hindrance. This is mainly due to the SAH-8 stapled peptide possessing a 2 amino acid extension compared to M06.

In addition to the differences in the packing arrangements made by the staple moieties of SAH-8 and M06 with Mdm2, there are significant changes in how the C-terminal ends of both peptides interact with the peptide binding groove (Figure 5). The SAH-8 stapled peptide is extended by two amino acids compared to M06. In the SAH-8:Mdm2 structure the Y100 of Mdm2 points into the pocket, stabilized by hydrogen bond interactions with N29. This results in occlusion of the pocket, and in turn shields L26 from solvent. This orientation of Y100 has been previously designated the “closed” conformation [27]. In contrast, the shorter helix of M06 does not fill the Mdm2 binding pocket as completely as SAH-8. This results in the ‘hinge’ region of Mdm2 adopting a helical conformation that caps the pocket, accommodating a snug fit of M06 into the cleft. The capping of the binding pocket by the Mdm2 ‘hinge’ helix is stabilized via hydrogen bond interactions between Q24 and the C-terminal amide of M06. It is clear that this movement of the ‘hinge’ can be accommodated by the Y100 either projecting into (‘closed’ conformation) or out (‘open’ conformation) of the Mdm2 binding pocket (Figure 5).

When Y100 adopts the ‘closed’ conformation, it forms a hydrogen bond with the backbone carbonyl of L26. I19 completes the formation of the M06 binding pocket by packing against Y100. In the ‘open’ conformation the ‘hinge’ helix and I19 are much more tightly packed against the Mdm2 binding pocket. This results in I19 occluding Y100 from orientating itself into the binding pocket and forcing it to adopt the ‘open’ position (Figure 5A). Interestingly, in the case of Nutlin, Y100 also orientates itself into the binding cleft to enclose it. However, it occupies an intermediate position between the two states observed in the Mdm2:M06 complexes. The I19 residue, instead of displacing Y100 or packing against it, is oriented above Y100 to form the Nutlin binding pocket. In addition, H96 interacts with the chloro-benzyl moiety of Nutlin, resulting in a swing of the helix (that contains H96) into the peptide binding groove, resulting in a narrowing of the pocket (Figure 5B); this feature is absent in the peptide complexes. These results further demonstrate the major role of the “gatekeeper” Y100 in mediating the remarkable plasticity in this region of the Mdm2 binding cleft [28]–[31], leading to accommodation of a variety of molecules ranging from the small to the increasingly bulky.

**Figure 6:**
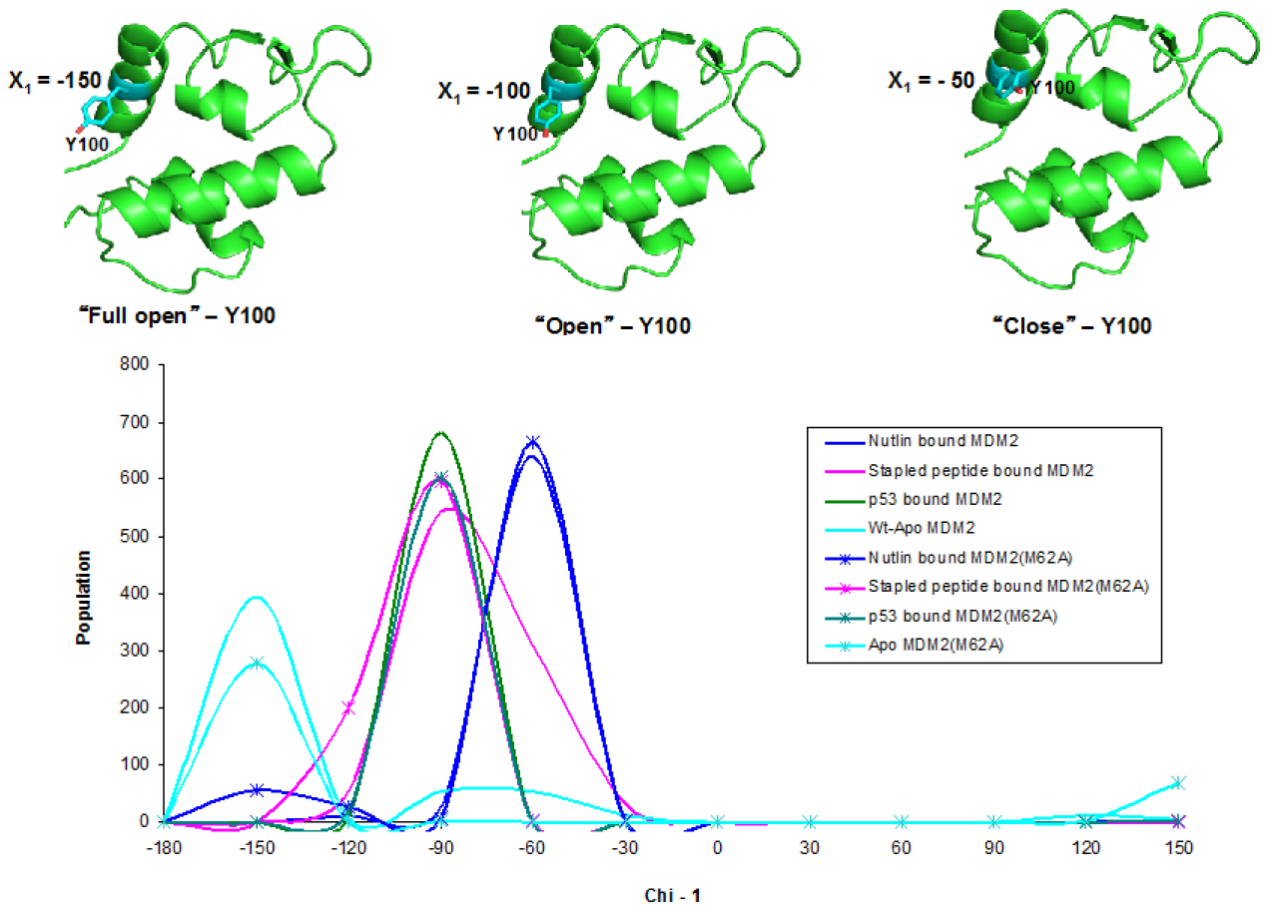
Flexibility of Y100 seen in Molecular Dynamics simulations of Mdm2. Molecular dynamics simulations (100 ns each) of Mdm2 in its apo, Nutlin-bound, p53 peptide-bound and stapled-peptide bound states shows that Y100 accesses 3 different conformational substrates as measured by the distribution of its chi1 torsional angle: a closed state that stabilizes Nutlin-like molecules, a fully open state that is seen in wild type and in the destabilized Nutlin-M62A complex, and an open state that stabilizes the peptides. Simulations were carried out as previously described [19].

The ‘open’ and ‘closed’ conformations of the Y100 residue have been respectively correlated with binding of either low or high affinity ligands [32]. The structures examined here reveal that the final conformation adopted by Y100 in the bound complex is additionally influenced by the size and bulk of the ligand (M06 and SAH-8 bind with relatively similar K_d_s of 127.7 nM and 50.4 nM, respectively). Furthermore, as can be seen with the structures of M06, the final bound conformation of Y100 appears to be flexible. This region of the Mdm2 binding pocket exhibits a large degree of plasticity, especially with regards to the “hinge” helix, which is displaced by the larger SAH-8 peptide but not by M06 or by Nutlin. Molecular dynamics simulations of Mdm2 in its apo, Nutlin, and peptide bound (both stapled and unstapled) forms shows that Y100 exists in 3 main states – a fully open, an open and a closed state (Figure 6). In wild type Mdm2, Y100 adopts open and fully open states which shift towards closed states upon Nutlin binding and open states in the presence of the p53 peptide. In comparison, the stapled peptide is associated with a broader distribution of Y100 states across the open state, stemming from the shorter length of the peptide compared to the p53 peptide. In the mutant, the weakening of Nutlin binding is seen by increased flexibility in Y100 including its shift to the fully open state that removes stabilizing contacts with Nutlin. In contrast, the p53 peptide remains stable while the adjustment to accommodate the stapled peptide is seen with a little shift towards a somewhat more open conformation in Y100. Additionally, when the pocket is formed on the Mdm2 surface, the individual packing arrangements of I19 and Y100 show a high degree of subtle conformational variations. The deep binding groove that forms in response to these binding events contrasts with the shallow interaction pocket observed in the NMR- derived apo structure (PDB: 1Z1M).

These new insights into the binding dynamics of the Mdm2 binding pocket should bring further perspective to rational design of new and more innovative Mdm2 agonists.

## Methods

### Peptide synthesis

The stapled peptide (M06) was synthesized by Anaspec (San Diego, Ca). The (*i, i* +7) hydrocarbon linkage was generated by placing the olefin-bearing unnatural amino acids (*S*)-2-(4′pentenyl) alanine and (*S*)-2-(7′-octenyl) alanine at positions 4 and 11, respectively. These were then fused via olefin metathesis using the Grubbs catalyst. M06 peptide was purified using HPLC to >90% purity. All peptides were amidated at their C-terminus and acetylated at their N-terminus.

### Protein Expression and Purification

Mdm2 (amino acids 6–125) was cloned as a GST-fusion protein using the pGEX-6P-1 GST expression vector (GE Healthcare). The mutant Mdm2-M62A (amino acids 6–125) was generated using QuickChange site directed mutagenesis protocol (Stratagene). The constructs were then transformed into *Escherichia coli* BL21(DE3) pLysS (Invitrogen) competent cells. Cells were grown in LB medium at 37°C and induced at OD_600 nm_ of 0.6 with 0.5 mM IPTG at 16°C. After overnight induction, the cells were harvested by centrifugation, resuspended in binding buffer (50 mM Tris-HCl pH 8.0, 150 mM NaCl), and lysed by sonication. After centrifugation for 60 mins at 19,000×g at 4°C, the cell lysate was then applied to a 5 mL GSTrap FF column (GE Healthcare) pre-equilibrated in wash buffer (50 mM Tris-HCl pH 8.0, 150 mM NaCl, 1 mM DTT). The proteins were cleaved on-column by PreScission protease (GE Healthcare) overnight at 4°C and eluted off the column with wash buffer. The protein sample was then dialyzed into buffer A solution (20 mM Bis-Tris, pH 6.5, 1 mM DTT) using HiPrep 26/10 Desalting column, and loaded onto a cation-exchange Resource S 1 mL column (GE Healthcare) pre-equilibrated in buffer A. The column was then washed in 6 column volumes of buffer A and bound protein was eluted with a linear gradient in buffer comprising 1 M NaCl, 20 mM Bis-Tris pH 6.5, and 1 mM DTT over 30 column volumes. Protein purity as assessed by SDS-PAGE was ∼95%, and the proteins were concentrated using Amicon-Ultra (3 kDa MWCO) concentrator (Millipore).

### Crystallization

The lyophilized stapled peptide M06 was first dissolved in DMSO to make a 25 mM stock solution. The purified proteins were concentrated to ∼2.5 mg/mL and then incubated with the stapled peptide at a 1∶3 molar ratio of protein to peptide at 4°C overnight. The sample was clarified by centrifugation before crystallization trials at 16°C using the sitting drop vapour diffusion method. Crystals of Mdm2-M62A in complex with peptide were obtained by mixing the protein-peptide complex with the reservoir solution in a ratio of 1∶1, with the reservoir solution containing 0.04 M Citric acid, 0.06 M Bis-Tris propane pH 6.4, and 20% PEG 3350.

### Data collection and Structure Determination

X-ray diffraction data was collected using synchrotron radiation beamline BL13B1 equipped with ADSC Quantum-315 CCD detector at the National Synchrotron Radiation Research Center (NSRRC, Taiwan). Data collection was carried out as a series of contiguous 1° oscillations for 170° rotations at a wavelength of 1.0 Å, exposure time at 5 s and the crystal to the detector distance at 280 mm. Data were indexed, integrated, and scaled using the HKL2000 program package. Structural determination was initiated by molecular replacement using the high-resolution human Mdm2 structure (PDB 2AXI) as a model in PHASER [33]. Restrained refinement with TLS was performed using REFMAC 5.0 [34] in the CCP4 suite of programs [35]. Model building was carried out in COOT [36]. The geometric restraints for the non natural amino acids ((*S*)-2-(4′pentenyl) alanine) and ((*R*)-2-(7'octenyl) alanine) (which form the hydrocarbon staple in sTIP-04) and the covalent bond linking their respective side-chains together to form the macrocyclic linkage were defined and generated using JLigand [37]. All structural figures were generated using PyMOL (Delano Scientific LLC). Validation of the final refined structures was performed using MOLPROBITY [38]. The atomic coordinates of the crystal structure have been deposited into the Protein Data Bank (PDB access code 4UMN).

### Fluorescence anisotropy

Fluorescence anisotropy assays were performed as previously described [18]. Titrations of purified wild-type and mutant Mdm2 (6–125) were incubated with 50 nM of carboxyfluorescein (FAM) labelled 12-1 peptide (FAM-RFMDYWEGL-NH2) to first determine the dissociation constants for the peptide-protein interaction. Apparent K_d_s of stapled peptide M06 were then determined by competitive fluorescence anisotropy. Titrations of M06 were carried out with a constant concentration of wild-type and mutant Mdm2 at 150 nM and the labelled peptide at 50 nM. Anisotropy measurements were carried out using the Envision Multilabel Reader (PerkinElmer). All experiments were carried out in PBS (2.7 mM KCl, 137 mM NaCl, 10 mM Na_2_HPO_4_ and 2 mM KH_2_PO_4_, pH 7.4), 3% DMSO and 0.1% Tween-20 buffer. All titrations were carried out 3–5 times. Curve fitting was carried out using Prism 4.0 (GraphPad).

### p53 reporter assay in T22 cells

All cells were maintained in Dulbecco’s Minimal Eagle Medium (DMEM) with 10% fetal bovine serum (FBS) and penicillin/streptomycin. T22 cells stably transfected with a p53 responsive β galactosidase reporter [39], were seeded into 96-well plates (8000 cells per well). After 24 hours incubation, cells were treated with compounds/peptide for 18 hours in DMEM with 10% FBS. β galactosidase activity was measured using the FluoReporter LacZ/Galactosidase Quantitation kit (Invitrogen) as per manufacturer’s instructions. Measurements were carried out on a Safire II multiplate reader (TECAN). All experiments were carried out in duplicate.

## Supporting Information

File S1 **Figures S1–S3. Figure S1. Comparitive binding of MO6 stapled peptide to Mdm2 (6–125) and Mdm2-M62A (6–125) as measured by flourescence anisotropy assay.** Competition titrations of MO6 stapled peptide against FAM-labelled p53-binding peptide 12.1 for binding to Mdm2 (6–125) (blue) and Mdm2-M62A (6–125) (purple). Values represent mean ± SD (n = 2) of a representative experiment. Final K_d_ values determined from average of 3–5 independent experiments. **Figure S2. T22 reporter assay measuring p53 transactivation.** Cells were treated with indicated Mdm2 ligands and p53 activity determined by measuring β galactosidase levels. Activity is expressed as fold increase over cells treated with vehicle only. Values represent average ± SD (n = 2). **Figure S3. Nutlin binding comprised by M62A mutation in Mdm2.** Structure of Nutlin (magenta) bound to Mdm2 (left) with M62 highlighted in yellow (adapted from 1YCR). When docked onto the structure of Mdm2-M62A (right), mutation to alanine (depicted in cyan) results in loss of significant packing interface and weak binding.
(PDF)

## References

[1] Kastan MB, Onyekwere O, Sidransky D, Vogelstein B, Craig RW (1991) Participation of p53 protein in the cellular response to DNA damage. Cancer Res 51: 6304–6311. 1933891

[2] Kuerbitz SJ, Plunkett BS, Walsh WV, Kastan MB (1992) Wild-type p53 is a cell cycle checkpoint determinant following irradiation. Proc Natl Acad Sci U S A 89: 7491–7495. 1323840 10.1073/pnas.89.16.7491 PMC49736

[3] Beroud C, Soussi T (1997) p53 and APC gene mutations: software and databases. Nucleic Acids Res 25: 138. 9016523 10.1093/nar/25.1.138 PMC146392

[4] Kan Z, Jaiswal BS, Stinson J, Janakiraman V, Bhatt D, et al (2010) Diverse somatic mutation patterns and pathway alterations in human cancers. Nature 466: 869–873. 20668451 10.1038/nature09208

[5] Oliner JD, Kinzler KW, Meltzer PS, George DL, Vogelstein B (1992) Amplification of a gene encoding a p53-associated protein in human sarcomas. Nature 358: 80–83. 1614537 10.1038/358080a0

[6] Bond GL, Hu W, Bond EE, Robins H, Lutzker SG, et al (2004) A single nucleotide polymorphism in the MDM2 promoter attenuates the p53 tumor suppressor pathway and accelerates tumor formation in humans. Cell 119: 591–602. 15550242 10.1016/j.cell.2004.11.022

[7] Momand J, Zambetti GP, Olson DC, George D, Levine AJ (1992) The mdm-2 oncogene product forms a complex with the p53 protein and inhibits p53-mediated transactivation. Cell 69: 1237–1245. 1535557 10.1016/0092-8674(92)90644-r

[8] Haupt Y, Maya R, Kazaz A, Oren M (1997) Mdm2 promotes the rapid degradation of p53. Nature 387: 296–299. 9153395 10.1038/387296a0

[9] Kubbutat MH, Jones SN, Vousden KH (1997) Regulation of p53 stability by Mdm2. Nature 387: 299–303. 9153396 10.1038/387299a0

[10] Honda R, Tanaka H, Yasuda H (1997) Oncoprotein MDM2 is a ubiquitin ligase E3 for tumor suppressor p53. FEBS Lett 420: 25–27. 9450543 10.1016/s0014-5793(97)01480-4

[11] Bernal F, Tyler AF, Korsmeyer SJ, Walensky LD, Verdine GL (2007) Reactivation of the p53 tumor suppressor pathway by a stapled p53 peptide. J Am Chem Soc 129: 2456–2457. 17284038 10.1021/ja0693587 PMC6333086

[12] Liu M, Li C, Pazgier M, Mao Y, Lv Y, et al (2010) D-peptide inhibitors of the p53-MDM2 interaction for targeted molecular therapy of malignant neoplasms. Proc Natl Acad Sci U S A 107: 14321–14326. 20660730 10.1073/pnas.1008930107 PMC2922601

[13] See HY, Lane DP (2010) A novel unstructured scaffold based on 4EBP1 enables the functional display of a wide range of bioactive peptides. J Mol Biol 404: 819–831. 20932973 10.1016/j.jmb.2010.09.063

[14] Shangary S, Wang S (2009) Small-molecule inhibitors of the MDM2-p53 protein-protein interaction to reactivate p53 function: a novel approach for cancer therapy. Annu Rev Pharmacol Toxicol 49: 223–241. 18834305 10.1146/annurev.pharmtox.48.113006.094723 PMC2676449

[15] Vassilev LT, Vu BT, Graves B, Carvajal D, Podlaski F, et al (2004) In vivo activation of the p53 pathway by small-molecule antagonists of MDM2. Science 303: 844–848. 14704432 10.1126/science.1092472

[16] Wei SJ, Joseph T, Sim AY, Yurlova L, Zolghadr K, et al (2013) In vitro selection of mutant HDM2 resistant to Nutlin inhibition. PLoS One 8: e62564. 23653682 10.1371/journal.pone.0062564 PMC3641235

[17] Kussie PH, Gorina S, Marechal V, Elenbaas B, Moreau J, et al (1996) Structure of the MDM2 oncoprotein bound to the p53 tumor suppressor transactivation domain. Science 274: 948–953. 8875929 10.1126/science.274.5289.948

[18] Brown CJ, Quah ST, Jong J, Goh AM, Chiam PC, et al. (2012) Stapled Peptides with Improved Potency and Specificity That Activate p53. ACS Chem Biol. 10.1021/cb3005148 23214419

[19] Wei SJ, Joseph T, Chee S, Li L, Yurlova L, et al (2013) Inhibition of nutlin-resistant HDM2 mutants by stapled peptides. PLoS One 8: e81068. 24278380 10.1371/journal.pone.0081068 PMC3835680

[20] Walensky LD, Kung AL, Escher I, Malia TJ, Barbuto S, et al (2004) Activation of apoptosis in vivo by a hydrocarbon-stapled BH3 helix. Science 305: 1466–1470. 15353804 10.1126/science.1099191 PMC1360987

[21] Kim YW, Grossmann TN, Verdine GL (2011) Synthesis of all-hydrocarbon stapled alpha-helical peptides by ring-closing olefin metathesis. Nat Protoc 6: 761–771. 21637196 10.1038/nprot.2011.324

[22] Verdine GL, Walensky LD (2007) The challenge of drugging undruggable targets in cancer: lessons learned from targeting BCL-2 family members. Clin Cancer Res 13: 7264–7270. 18094406 10.1158/1078-0432.CCR-07-2184

[23] Phillips C, Roberts LR, Schade M, Bazin R, Bent A, et al (2011) Design and structure of stapled peptides binding to estrogen receptors. J Am Chem Soc 133: 9696–9699. 21612236 10.1021/ja202946k

[24] Pazgier M, Liu M, Zou G, Yuan W, Li C, et al (2009) Structural basis for high-affinity peptide inhibition of p53 interactions with MDM2 and MDMX. Proc Natl Acad Sci U S A 106: 4665–4670. 19255450 10.1073/pnas.0900947106 PMC2660734

[25] Baek S, Kutchukian PS, Verdine GL, Huber R, Holak TA, et al (2012) Structure of the stapled p53 peptide bound to Mdm2. J Am Chem Soc 134: 103–106. 22148351 10.1021/ja2090367

[26] Anil B, Riedinger C, Endicott JA, Noble ME (2013) The structure of an MDM2-Nutlin-3a complex solved by the use of a validated MDM2 surface-entropy reduction mutant. Acta Crystallogr D Biol Crystallogr 69: 1358–1366. 23897459 10.1107/S0907444913004459

[27] Popowicz GM, Czarna A, Rothweiler U, Szwagierczak A, Krajewski M, et al (2007) Molecular basis for the inhibition of p53 by Mdmx. Cell Cycle 6: 2386–2392. 17938582 10.4161/cc.6.19.4740

[28] Dastidar SG, Lane DP, Verma CS (2008) Multiple peptide conformations give rise to similar binding affinities: molecular simulations of p53-MDM2. J Am Chem Soc 130: 13514–13515. 18800837 10.1021/ja804289g

[29] Dastidar SG, Lane DP, Verma CS (2009) Modulation of p53 binding to MDM2: computational studies reveal important roles of Tyr100. BMC Bioinformatics 10 Suppl 15S6. 10.1186/1471-2105-10-S15-S6 PMC2788357 19958516

[30] Schon O, Friedler A, Bycroft M, Freund SM, Fersht AR (2002) Molecular mechanism of the interaction between MDM2 and p53. J Mol Biol 323: 491–501. 12381304 10.1016/s0022-2836(02)00852-5

[31] Kallen J, Goepfert A, Blechschmidt A, Izaac A, Geiser M, et al (2009) Crystal Structures of Human MdmX (HdmX) in Complex with p53 Peptide Analogues Reveal Surprising Conformational Changes. J Biol Chem 284: 8812–8821. 19153082 10.1074/jbc.M809096200 PMC2659239

[32] Brown CJ, Dastidar SG, Quah ST, Lim A, Chia B, et al (2011) C-terminal substitution of MDM2 interacting peptides modulates binding affinity by distinctive mechanisms. PLoS One 6: e24122. 21904608 10.1371/journal.pone.0024122 PMC3164098

[33] McCoy AJ, Grosse-Kunstleve RW, Adams PD, Winn MD, Storoni LC, et al (2007) Phaser crystallographic software. J Appl Crystallogr 40: 658–674. 19461840 10.1107/S0021889807021206 PMC2483472

[34] Murshudov GN, Vagin AA, Dodson EJ (1997) Refinement of macromolecular structures by the maximum-likelihood method. Acta Crystallogr D Biol Crystallogr 53: 240–255. 15299926 10.1107/S0907444996012255

[35] Winn MD, Ballard CC, Cowtan KD, Dodson EJ, Emsley P, et al (2011) Overview of the CCP4 suite and current developments. Acta Crystallogr D Biol Crystallogr 67: 235–242. 21460441 10.1107/S0907444910045749 PMC3069738

[36] Emsley P, Lohkamp B, Scott WG, Cowtan K (2010) Features and development of Coot. Acta Crystallogr D Biol Crystallogr 66: 486–501. 20383002 10.1107/S0907444910007493 PMC2852313

[37] Lebedev AA, Young P, Isupov MN, Moroz OV, Vagin AA, et al (2012) JLigand: a graphical tool for the CCP4 template-restraint library. Acta Crystallogr D Biol Crystallogr 68: 431–440. 22505263 10.1107/S090744491200251X PMC3322602

[38] Chen VB, Arendall WB 3rd, Headd JJ, Keedy DA, Immormino RM, et al (2010) MolProbity: all-atom structure validation for macromolecular crystallography. Acta Crystallogr D Biol Crystallogr 66: 12–21. 20057044 10.1107/S0907444909042073 PMC2803126

[39] Lu X, Burbidge SA, Griffin S, Smith HM (1996) Discordance between accumulated p53 protein level and its transcriptional activity in response to u.v. radiation. Oncogene 13: 413–418. 8710381

