# Supplementary material for: Structure of a Stapled Peptide Antagonist Bound to Nutlin-Resistant Mdm2
Source: PLoS One. 2014 Aug 12;9(8):e104914. doi: 10.1371/journal.pone.0104914 (PMC4130638; doi:10.1371/journal.pone.0104914)
Supplement: File S1 — Figures S1–S3. Figure S1. Comparitive binding of MO6 stapled peptide to Mdm2 (6–125) and Mdm2-M62A (6–125) as measured by flourescence anisotropy assay. Competition titrations of MO6 stapled peptide against FAM-labelled p53-binding peptide 12.1 for binding to Mdm2 (6–125) (blue) and Mdm2-M62A (6–125) (purple). Values represent mean ± SD (n = 2) of a representative experiment. Final Kd values determined from average of 3–5 independent experiments. Figure S2. T22 reporter assay measuring p53 transactivation. Cells were treated with indicated Mdm2 ligands and p53 activity determined by measuring β galactosidase levels. Activity is expressed as fold increase over cells treated with vehicle only. Values represent average ± SD (n = 2). Figure S3. Nutlin binding comprised by M62A mutation in Mdm2. Structure of Nutlin (magenta) bound to Mdm2 (left) with M62 highlighted in yellow (adapted from 1YCR). When docked onto the structure of Mdm2-M62A (right), mutation to alanine (depicted in cyan) results in loss of significant packing interface and weak binding. (PDF) [file pone.0104914.s001.pdf]

Figure S1

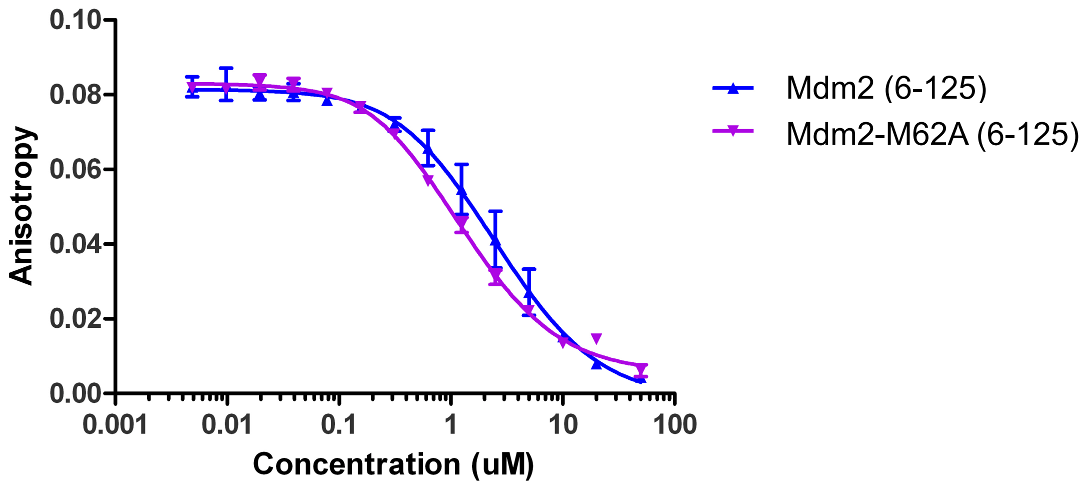

Figure S2

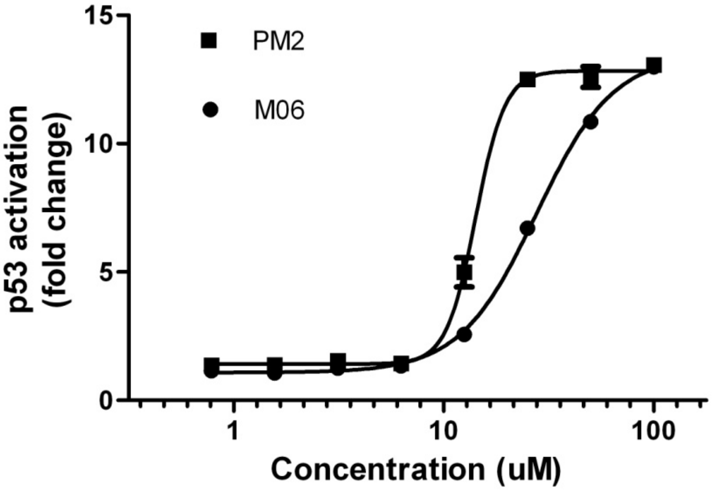

Figure S3

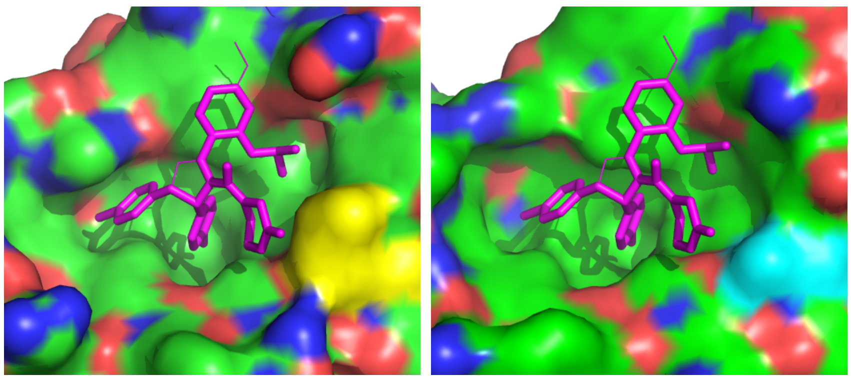

Figure **S1,** Comparitive binding of MO6 stapled peptide to Mdm2 (6-125) and Mdm2-M62A (6-125) as measured by flourescence anisotropy assay. Competition titrations of MO6 stapled peptide against FAM-labelled p53-binding peptide 12.1 for binding to Mdm2 (6-125) (blue) and Mdm2-M62A (6-125) (purple). Values represent mean ± SD (n=2) of a representative experiment. Final K_d values determined from average of 3-5 independent experiments. Figure **S2**, T22 reporter assay measuring p53 transactivation. Cells were treated with indicated Mdm2 ligands and p53 activity determined by measuring β galactosidase levels. Activity is expressed as fold increase over cells treated with vehicle only. Values represent average ± SD (n=2). Figure **S3,** Nutlin binding comprised by M62A mutation in Mdm2. Structure of Nutlin (magenta) bound to Mdm2 (left) with M62 highlighted in yellow (adapted from 1YCR). When docked onto the structure of Mdm2-M62A (right), mutation to alanine (depicted in cyan) results in loss of significant packing interface and weak binding.
